# Recognition of and Response to Neonatal Intrapartum-related Complications in Home-birth Settings in Bangladesh

**Published:** 2014-09

**Authors:** Kristin E. VanderEnde, Allisyn C. Moran, Karen Leasure, Louise T. Day, Kaosar Afsana, Nahid Kalim, Shirajum Munira, Nazneen Rahman, Munia Islam, Jasmin Khan, Lynn M. Sibley

**Affiliations:** ^1^Hubert Department of Global Health, Emory University, USA; ^2^Reproductive Health Unit, icddr,b, Mohakhali, Dhaka 1212, Bangladesh; ^3^Nell Hodgson Woodruff School of Nursing, Emory University, USA; ^4^MIS-Research Department, LAMB, Bangladesh; ^5^BRAC Health Program, BRAC, Dhaka, Bangladesh; ^6^James P. Grant School of Public Health, BRAC University, Dhaka, Bangladesh

**Keywords:** Home-birth, Intrapartum-related complications, Bangladesh

## Abstract

Intrapartum-related complications (previously called ‘birth asphyxia’) are a significant contributor to deaths of newborns in Bangladesh. This study describes some of the perceived signs, causes, and treatments for this condition as described by new mothers, female relatives, traditional birth attendants, and village doctors in three sites in Bangladesh. Informants were asked to name characteristics of a healthy newborn and a newborn with difficulty in breathing at birth and about the perceived causes, consequences, and treatments for breathing difficulties. Across all three sites ‘no movement’ and ‘no cry’ were identified as signs of breathing difficulties while ‘prolonged labour’ was the most commonly-mentioned cause. Informants described a variety of treatments for difficulty in breathing at birth, including biomedical and, less often, spiritual and traditional practices. This study identified the areas that need to be addressed through behaviour change interventions to improve recognition of and response to intrapartum-related complications in Bangladesh.

## INTRODUCTION

Worldwide, more than three million newborn babies die (1), and another three million babies are stillborn (2) each year. The vast majority (>98%) of both neonatal deaths and stillbirths occur in low- and middle-income countries (3-4). While other causes of under-five mortality are decreasing, neonatal deaths account for 40% of deaths in children below the age of five years, with intrapartum-related complications (previously called ‘birth asphyxia’) being a significant contributor to under-five mortality (9.4% or 0.7 million) (5). In addition, there is a significant burden of impairment due to intrapartum-related complications, including the development of cerebral palsy, learning difficulties, or other disabilities (6). Risk factors for intrapartum-related complications, defined for our purposes as an infant with perinatal respiratory depression after birth, include prematurity, low birthweight, intrauterine growth restriction, and antepartum (e.g. anaemia, eclampsia) and intrapartum (e.g. prolonged labour, umbilical cord prolapse) complications (6).

In South Asia, an even greater proportion (52.3%) of under-five deaths occurs during the neonatal period, with 10% of all under-five deaths attributed to intrapartum-related complications (5). In Bangladesh alone, this translates into a yearly estimate of 83,070 babies dying in their first 28 days of life, and of them, 19,172 dying from intrapartum-related complications (5). The majority of these babies are born and die in the home setting (7). Unskilled or low-skilled birth attendants, such as family members and traditional birth attendants (TBAs), who often assist women delivering at home in Bangladesh, may not have the skills to recognize and respond to intrapartum-related complications in a newborn. This highlights a discontinuity between the onset of the problem, recognition and appropriate healthcare-seeking. In the case of intrapartum-related complications, timely recognition and response is crucial as even a few minutes delay in seeking and receiving appropriate care may result in substantial injury to the newborn and may represent the difference between life and death (6).

This paper focuses on the first stage of a three-phase, three-site study on prolonged labour and intrapartum-related complications in home-birth settings in Bangladesh. This study was a collaborative work between Emory University and three internationally-recognized organizations in Bangladesh: icddr,b, BRAC, and LAMB Integrated Rural Health and Development. The objective of this study was to understand how new mothers, female relatives, traditional birth attendants, and village doctors recognize and respond to a newborn with difficulty in breathing after birth in home-birth settings in three sites in Bangladesh.

## MATERIALS AND METHODS

In this paper, we present the first stage of a three-phase study, an analysis of interviews with new mothers, female relatives, TBAs, and village doctors (n=240) in one urban and two rural sites in Bangladesh. In this analysis, we used successive freelisting techniques to elicit (i) how informants in these three sites recognized a healthy newborn and a newborn with difficulty in breathing at birth and (ii) informants’ descriptions of the causes, consequences, and care practices for a newborn with such breathing difficulties.

### Setting

The study was conducted at two rural sites (Matlab and LAMB) and one urban site (BRAC) in Bangladesh. Matlab Health Research Centre of icddr,b in Matlab, Chandpur district, is located in a densely-populated rural area of Bangladesh. Beginning in 1966, icddr,b has been continually operating a Health and Demographic Surveillance System (HDSS) for a rural population of approximately 220,000 and has been operating a Maternal, Child Health and Family Planning programme since 1978. Four subcentres provide 24-hour nursing and midwifery services. In 2007, forty-four percent of births took place at home, and the neonatal mortality rate was 20.6 per 1,000 livebirths, with 29% of these deaths attributed to intrapartum-related complications.

The urban site was based in two large slum areas in the capital city Dhaka. BRAC, established in 1972, is one of the largest non-governmental organizations in the world, running community programmes focused on poverty alleviation and empowerment of the poor in rural and urban slum areas in Bangladesh and around the world. In 2007, BRAC initiated *Manoshi*, a community-based intervention aimed at reducing maternal, neonatal, and child death and addressing other diseases in urban slums in Dhaka. The two largest slum areas—Korail (3 delivery centres) and Kamrangir Char (7 delivery centres) were chosen as the sites for the study. In 2006, the neonatal mortality rate in Dhaka's urban slums was 43.7 per 1,000 livebirths (8). In 2007, eighty-five percent of births in the *Manoshi* project area took place at home (9). In 2008-2009, forty-eight percent of neonatal deaths in the *Manoshi* project area were attributed to intrapartum-related complications (10).

The third site studied, LAMB, is located in rural northwest Bangladesh. LAMB Integrated Rural Health and Development in Dinajpur district has worked towards equitable community health and development, with a specific focus on women and children since 1976. LAMB Community Health and Development (CHDP) serves a population of approximately one million, with LAMB Hospital serving as a referral centre for a broader population of 2 to 2.5 million. From the LAMB CHDP area, four geographical areas (unions), with home-birth rates ranging from 65% to 92%, were selected. Data from verbal autopsy showed that, in 2007, the average perinatal mortality rate was approximately 48 per 1,000 births, and 41% of early neonatal deaths were attributed to intrapartum-related complications.

This was the first phase of a larger study funded by Emory University Global Health Institute, focusing on recognition of and response to prolonged labour and birth asphyxia.

### Sample

To ensure the breadth and depth of terms and concepts, we obtained a sample of 100 informants at each site—20 each from five different groups that are important in the home-birth setting. They included: (i) women of reproductive age who gave birth in 2007; (ii) older female relatives (aged 50-70 years) living in the extended family and potentially influential in matters relating to childbirth (elder influential women); (iii) traditional birth attendants (TBA); (iv) village doctors (medically untrained allopathic practitioners); and (v) skilled birth attendants (SBA) who provided care at local hospitals and subcentre clinics. The sample-size for each subgroup was calculated based on an assumed low level of cultural sharing (0.50), and high degrees of accuracy (0.95) and confidence (0.95) (11-13). We identified informants through the existing community health and demographic and surveillance systems and lists of local healthcare providers. In this article, we focus on the signs, causes, and care practices that would occur before seeking care outside the home. For this reason, we excluded the SBA group from the following analysis and focus on respondents who would be most likely to provide and make decisions regarding care inside the home.

### Data collection

The study protocol was approved by Emory University's Institutional Review Board, the Ethical Review Committee of icddr,b and the LAMB Research Ethics Committee. The BRAC Management Technical Committee reviewed and approved the proposal, and the study was allowed to proceed after ethical review was approved by icddr,b. Trained interviewers obtained voluntary verbal informed consent from all informants.

The research team developed a semi-structured questionnaire to elicit local words and short phrases associated with newborns (healthy and sick). The questionnaire consisted of open-ended questions to elicit spontaneous responses, using successive freelisting, a technique that asks informants to list all the local terms (or items) they know in a particular domain (14). For the current research, respondents were asked for signs of a ‘healthy newborn baby’ and a ‘newborn with difficulty in breathing at birth’, followed by questions on causes, consequences, and care practices for a newborn with difficulty in breathing at birth. The questionnaire, which also included questions to elicit standard demographic and social information as well as experience with childbirth, was translated and back-translated, pretested and revised before use.

At each site, there were at least two trained interviewers who conducted face-to-face interviews in the local language Bangla at a time and place convenient for the informant. Many of the interviewers had a background in social science, and all received training from the study investigators. During data collection, quality was ensured through verification at the conclusion of each interview, daily debriefing about interviews among study teams, and communication among team leaders and principal investigators, if questions arose. In addition, each interview was audio-recorded, and interviewers recorded informants’ verbatim responses directly against the completed questionnaire.

### Data analysis

Information in the completed forms was transcribed and translated into English and uploaded into EZ-text, a software for qualitative data management (15). Using an initial list of codes based on the informant, two coders based at Emory University (KV and KL) individually sorted the codes into broad categories, identified synonyms, and eliminated direct duplicates within categories. The coders, who were both graduate students at the time of the study and were trained and supervised by one of the authors (LS), then met to compare drafts. Differences were discussed, and codes were combined, renamed, or eliminated accordingly. This process was repeated two more times, at which point it was felt that all relevant codes had been identified. Each of the identified codes was given an 8-character EZ-text code, and the first draft of the codebook was entered into EZ-text.

Following the initial development, three rounds of coding were used for further refining the codebook, with inter-coder reliability statistics (kappa) calculated for each round. At this point, the two coders coded the entire set of responses from one site—Matlab (n=100)—with a reliability check taking place at the midpoint and completion of coding. Upon completion of coding, discrepancies were discussed and resolved by mutual agreement. The process was repeated for the responses from BRAC and LAMB, with the final codebook developed, tested, and refined across all three sites.

We retained all codes which had high inter-rater reliability (kappa >0.8) and that were also mentioned by >20% of informants (for at least one site) or were of theoretical interest. We summarized frequently-mentioned signs of both a healthy newborn and a newborn with breathing difficulties and the causes and care practices associated with such a baby across all three sites in tables and charts. In analyzing similarities and differences across sites, we also used quotes from respondents to emphasize and clarify the quantitative findings.

Throughout the study, deliberate steps were taken to ensure that results were culturally relevant and meaningful. These protections included close communication between independent coders located in the United States and local research teams of native Bangla speakers at each site. Other quality control measures included a glossary of local terms to aid in translation and coding and frequent inter-rater reliability checks during coding.

## RESULTS

### Characteristics of informants

In all three sites, the majority of informants were married and Muslim. All of the village doctors were men, and the other informants (women of reproductive age, elder influential women, and TBAs) were all women. Women of reproductive age were younger, had lower parity, and reported receiving higher levels of education than both elder influential women and the TBAs. Across all sites, the male village doctors reported the highest levels of formal education. Informants from the BRAC site, located in Dhaka, reported higher income than informants in rural areas (Table).

### Descriptions of a healthy newborn and a newborn with difficulty in breathing at birth

Informants were asked to describe the characteristics or the signs of a healthy newborn and a newborn with difficulty in breathing at birth. Specifically, informants were asked to describe the breathing pattern, colour, crying, movement, and other signs, indicating either a healthy newborn or a newborn with breathing difficulties. Informants depicted the breathing of a healthy newborn with a number of non-specific signs, including deep breathing, rapid breathing, indeterminate breathing, and most commonly, slow breathing. While a majority of informants from BRAC (75%) and Matlab (61%) stated that a healthy newborn had slow breathing, this answer was less common for respondents from LAMB (38%). In fact, slow breathing was the most commonly-identified sign of difficulty in breathing at birth for informants from the LAMB site (59%). The most frequently-mentioned descriptions of a newborn with difficulty in breathing at birth varied between sites, with ‘no breathing’ highest for the BRAC site (65%) and ‘gasping’ highest for Matlab (48%).

Across all sites, informants were fairly consistent in their characterization of the sounds and movements of healthy newborns and newborns with breathing difficulties. Healthy newborns cry (≥64% of informants across all sites) and move vigorously (≥95%) while newborns with breathing problems have no cry (≥54%) and no movement (≥65%).

In general, whitish colour was associated with a healthy baby, with over 60% of informants mentioning this in all sites. Blackish colour was most frequently associated with a baby with difficulty in breathing at birth (≥49% of informants across all sites). The reverse was also common, as in two locations (BRAC and Matlab), a sizeable number of informants (over 20%) in each site cited blackish colour as a sign of a healthy baby while 19% of informants at LAMB and BRAC and 20% at Matlab cited whitish colour as a sign of a baby with breathing difficulties.

### Perceived causes of difficulty in breathing at birth

**Figure 1.**
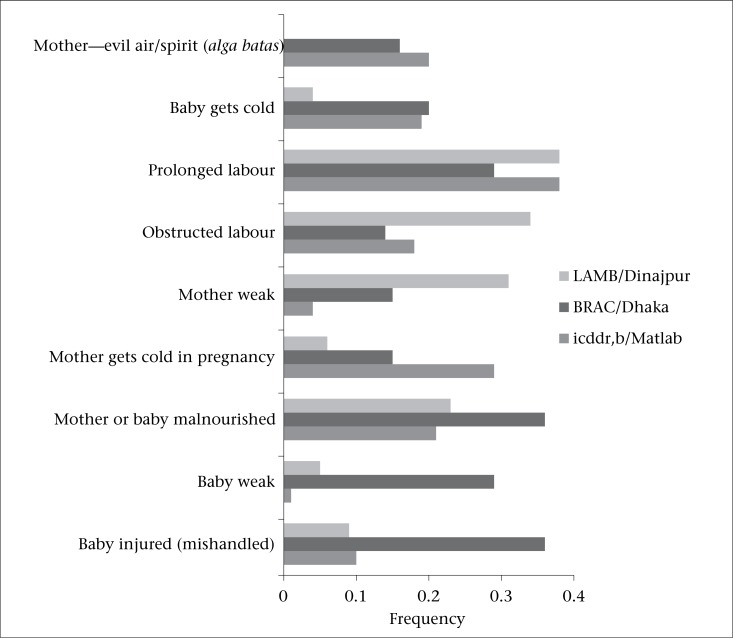
Perceived causes of difficulty in breathing at birth

Perceived causes of difficulty in breathing at birth include biomedical explanations, such as prolonged and obstructed labour, injury, and malnourishment, or weakness of the mother or the baby. Additionally, it was also attributed to more traditional or spiritual causes, including evil air/spirit (*alga batas*), and the mother or the baby getting cold during pregnancy (Figure 1). These perceived causes are described in greater detail below.

Thirty-eight percent of informants from Matlab and LAMB and 29% from BRAC identified prolonged labour as a cause:

The mother has less labour pain so that she takes a long time to deliver, and the baby has difficulty in breathing (elder influential woman, LAMB).

More informants from LAMB (34%) mentioned obstructed labour (the baby stuck inside the birth canal) as a cause of breathing difficulties at birth compared to informants from BRAC (14%) or Matlab (18%). Across all sites, informants were consistent in their description of the baby getting ‘stuck’ in the mother's uterus. A woman who gave birth in 2007 recounted:

The baby can't take breath, when it becomes stuck at mother's mouth of uterus (woman of reproductive age, BRAC).

An informant from the LAMB site echoed this view:

The baby is stuck in the mouth of the uterus because the mouth of the uterus is narrow; the baby can't come out easily, they have difficulty in breathing (TBA, LAMB).

Informants perceived that injury to the baby, including mishandling, frequent vaginal exams, rotating the child, mismanagement of labour, and injury in mother's abdomen could cause breathing problems. This belief, while expressed across all sites, was a prominent response among informants from BRAC (36%) compared to the LAMB site (9%) and Matlab (10%):

**Table.** Characteristics of informants

Characteristics	LAMB/Dinajpur (n= 80)				BRAC/Dhaka (n=80)				icddr,b/Matlab (n=80)			
	WRA	EIW	TBA	VD	WRA	EIW	TBA	VD	WRA	EIW	TBA	VD
Sample-size	20	20	20	20	20	20	20	20	20	20	20	20
Mean age in years	21.0	53.8	42.5	42.5	24.7	56.1	52.1	36.9	27.1	59.4	55.8	46.8
(SD)	(3.5)	(6.2)	(10.5)	(11.8)	(4.9)	(6.7)	(9.4)	(13.5)	(6.0)	(6.7)	(7.1)	(11.9)
Mean parity	1.9	6.1	4.7	NA*	3.0	6.5	7.3	NA*	2.1	6.6	5.5	NA*
(SD)	(0.97)	(2.1)	(2.1)	NA*	(2.2)	(2.0)	(4.0)	NA*	(1.1)	(2.5)	(1.8)	NA*
Educational level (%)												
No schooling	5.0	80.0	30.0	0.0	35.0	70.0	65.0	0.0	0.0	55.0	55.0	0.0
Primary	30.0	20.0	60.0	0.0	35.0	25.0	25.0	0.0	40.0	40.0	35.0	0.0
Secondary	65.0	0.0	10.0	40.0	30.0	5.0	10.0	35.0	35.0	5.0	10.0	60.0
College	0.0	0.0	0.0	55.0	0.0	0.0	0.0	40.0	20.0	0.0	0.0	30.0
Graduate school	0.0	0.0	0.0	5.0	0.0	0.0	0.0	25.0	5.0	0.0	0.0	10.0
Income (Tk) (%)												
<2,500	35.0	45.0	55.0	15.0	0.0	5.0	0.0	0.0	5.0	25.0	30.0	5.0
2,500-5,000	55.0	45.0	30.0	65.0	35.0	5.0	35.0	5.0	35.0	25.0	45.0	35.0
>5,000	10.0	10.0	15.0	20.0	65.0	90.0	65.0	95.0	60.0	50.0	25.0	60.0
Religion (%)												
Muslim	90.0	80.0	90.0	65.0	100.0	100.0	100.0	85.0	80.0	85.0	90.0	70.0
Hindu	5.0	20.0	10.0	35.0	0.0	0.0	0.0	15.0	20.0	15.0	10.0	30.0
Other	5.0	0.0	0.0	0.0	0.0	0.0	0.0	0.0	0.0	0.0	0.0	0.0
Marital status (%)												
Married	100.0	90.0	80.0	95.0	100.0	80.0	70.0	75.0	95.0	75.0	60.0	100.0
Widowed	0.0	10.0	20.0	0.0	0.0	20.0	30.0	0.0	0.0	25.0	35.0	0.0
Divorced	0.0	0.0	0.0	0.0	0.0	0.0	0.0	0.0	5.0	0.0	0.0	0.0
Separated	0.0	0.0	0.0	0.0	0.0	0.0	0.0	0.0	0.0	0.0	5.0	0.0
Never married	0.0	0.0	0.0	5.0	0.0	0.0	0.0	25.0	0.0	0.0	0.0	0.0

*Male respondents; EIW=Elder influential woman (>50 years of age); TBA=Traditional birth attendant; VD=Village doctor; WRA=Woman of reproductive age (15-49 years) who gave birth in 2007

During delivery, if the *dai* (home-based birth attendant) holds the baby's neck and pulls to make baby out from abdomen, the baby gets pressure and, after birth, the baby can't take breath (TBA, BRAC).

Across sites, informants attributed malnourishment and weakness of both mother and newborn as contributing to difficulty in breathing at birth:

If the mother cannot take food in her pregnancy period, then her baby has malnutrition, and it may have the breathing problem (elder influential woman, Matlab).

Due to the mother's lack of nutrition, meaning when she is pregnant, she didn't eat fruits properly; so, she has no strength, she can't push to deliver; so, the baby comes late; for this reason, they have difficulty in breathing (village doctor, LAMB).

Informants also perceived traditional and spiritual causes for difficulty in breathing at birth. Informants from Matlab (29%, 19%) and BRAC (15%, 20%), and to a lesser extent, from LAMB (6%, 4%) perceived coldness of either mother or baby (mother gets cold in pregnancy; baby gets cold, low temperature, not covered by cloth) as a cause:

Baby gets cold from mother. If mother [takes] bath late in the evening or eat cold rice, do not dry her hair for long time, the baby in the womb will get cold and cause breathing problem later (woman of reproductive age, Matlab).

If mother touches cold water several times during pregnancy, then the baby also gets cold and just after birth, that baby can't take breath (TBA, BRAC).

While not seen in informants’ responses from the LAMB site, informants from both BRAC (16%) and Matlab (20%) attributed breathing difficulties of the baby at birth to evil air or evil spirits:

If the evil air touches the mother when she goes outside in the mid-noon or night, her baby would have the breathing problem (elder influential woman, Matlab).

During pregnancy period if mother moves outside at dawn, then evil spirit comes and catches the baby's neck with pressure in mother's abdomen; then after birth, that baby can't take breath (woman of reproductive age, BRAC).

### Perceived treatments

**Figure 2.**
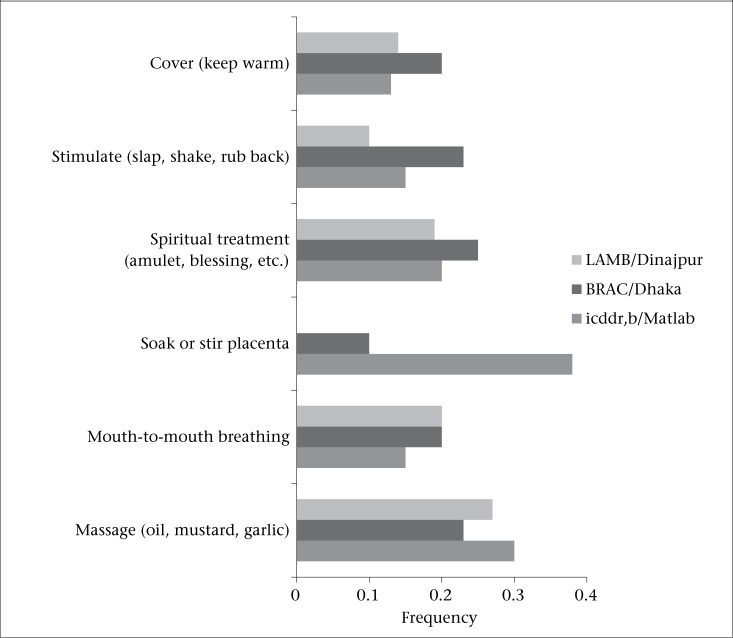
Perceived home treatments for difficulty in breathing at birth

Informants were asked to name the things that are done to help a newborn with difficulty in breathing at birth, both at home and in a health facility. As the focus of this analysis is on home-based care, we analyzed only the responses addressing home-based care practices. In addition to biomedical care practices (mouth-to-mouth breathing, stimulation, and warming or covering the baby), informants also mentioned traditional (massage with oil and soaking/stirring the placenta) and spiritual care practices (Figure 2).

No single care practice was mentioned by more than 40% of informants at one site. Soaking or stirring the placenta, the most frequently-mentioned home-based care practice for informants from Matlab (38%), was only mentioned by 10% of respondents from the BRAC site and none from the LAMB site. An elder influential woman explained the rationale for this care practice:

*Dai* puts the placenta into the cold water because then the breath comes to the baby … [because] if the placenta is made wet in the cold water, then the baby begins to take breath (elder influential woman, BRAC).

A village doctor said:

If the placenta is moved/stirred on warm water, then gradually, the baby starts to breathe. I also do this. I suggest others to do that. By this way, the baby was kept alive … [because] the baby can take oxygen from the placenta (village doctor, Matlab).

Informants across all three sites mentioned massaging the baby with oil, mustard, or garlic as a treatment:

Garlic and oil they heat up, and they massage their [baby's] hands and legs so that the baby will be warm and will get healthy (woman of reproductive age, LAMB).

In addition to these traditional treatments, informants in all sites described spiritual care practices, including exorcisms, blessed blows, and amulets intended to help the newborn breathe:

The traditional healer *kobiraj* do exorcism; so that the baby can take breath (elder influential woman, BRAC).

Give the baby blessed blow, amulets, blessed oil by a *fakir*; massage the blessed oil on her/his body… to save the baby from evil spirit (TBA, Matlab).

Biomedical treatments mentioned by informants included mouth-to-mouth breathing, covering or wrapping the baby in cloth, and stimulating the baby:

They blow in the baby's mouth so that the breath will come, and it will cry (elder influential woman, LAMB).

Wrap the baby so that baby remains warm. In this way, baby might start taking breath (village doctor, Matlab).

*Dai* gives pat on the back of baby …after giving pat, the baby starts crying. When baby cries, baby can take breath (woman of reproductive age, BRAC)

## DISCUSSION

The findings from this study indicate fairly consistent agreement in terms of signs, causes, and treatments for babies with difficulty in breathing at birth, with some variation among sites. These findings are important to focus the development of health education messages to identify babies with difficulty in breathing at birth and ensure appropriate care and treatment.

Across all sites, respondents reported not crying or moving at birth as a sign of a breathing problem. However, there was some variation among sites in terms of the rate of breathing. In Matlab and BRAC sites, slow breathing indicated a healthy baby while, in the LAMB site, slow breathing indicated both a healthy and an unhealthy baby. There was also discrepancy in terms of the colour of the baby. In all three sites, respondents differentiated between ‘white’ and ‘black’ colour of the baby. Some respondents perceived a ‘white-colour baby’ to be healthy, and others perceived the same baby to be unhealthy. The same finding was true for babies perceived to be ‘blackish’ in colour. The variation among respondents, both within and between sites, reflects the challenges in identifying and describing the breathing patterns and colour of both healthy and unhealthy newborns.

In terms of causes of breathing difficulties at birth, there was consistency among the sites. In all sites, especially in the LAMB site, prolonged or obstructed labour was perceived as a cause for a newborn with difficulty in breathing. Respondents in all sites also cited weakness or malnourishment on the part of the mother or baby as a cause. In BRAC, respondents cited poor labour practices as a cause; this was less commonly cited in the Matlab or LAMB sites. Poor labour practices included manipulation of the baby during birth, rotating the child, and injury to the woman's abdomen. Interestingly, evil spirits/air (*alga batas*) was cited as a cause of difficulty in breathing at Matlab and the BRAC site. While evil spirit was not cited in the LAMB site under questions relating to the cause of difficulty in breathing at birth, spiritual treatments for breathing difficulties (e.g. amulet, blessing) were described with similar frequency across all three sites. In South Asia, babies are perceived to be vulnerable to illness and susceptible to evil spirits in the first month of life (16-17). This perception of vulnerability often inhibits taking the baby outside the home for treatment for illness, to avoid being ‘caught’ by evil spirits/air. As a result, many newborns who are perceived to be ill are treated inside the home by unqualified providers (18). Our results suggest that these beliefs are strongly held across diverse geographical settings. In Dhaka, other studies in urban slum settings have indicated that newborn care-related beliefs and practices are similar to those in rural areas (19).

There was fairly a consistent agreement between the sites on treatment for difficulty in breathing at birth, which includes stimulation of the baby, covering/keeping baby warm, spiritual treatments, and mouth-to-mouth breathing. In Matlab, the belief around stirring or soaking the placenta was cited by almost 40% of the respondents while this was cited by only 10% of respondents in the BRAC site and none in the LAMB site. Another interesting finding is the prevalence of knowledge around mouth-to-mouth breathing to treat difficulty in breathing at birth. This was cited by 20% of respondents in the LAMB and BRAC sites and 15% of respondents in Matlab. Unfortunately, we did not collect information on how respondents described performing mouth-to-mouth breathing. It would be helpful to know if women or birth attendants actually performed mouth-to-mouth breathing, or only if they had knowledge as to its benefits. The evidence suggests that the community-based management of intrapartum-related complications (including mouth-to-mouth breathing) is feasible, especially in settings where home-based birth is the norm, and community-based cadres are available (20). However, several conditions need to be in place, including the presence of community-based providers at birth, training and supervision, and linkages with health systems to ensure equipment and supplies as well as referral, if needed. Several community-based trials are in process that will add to the evidence-base (20).

Overall, this study provides useful findings on how women, village doctors, and traditional birth attendants in three diverse sites in Bangladesh differentiate between healthy babies and those with perceived difficulty in breathing at birth. The findings demonstrate that there are common signs and symptoms to identify babies with difficulty in breathing at birth as well as causes and treatments. As a consequence of our focus on comparability across sites, we were limited in our ability to fully capture the complexities within each site, for instance, the differences between different categories of respondents, such as older and younger women. Additionally, while respondents from the LAMB site did not mention stirring or soaking the placenta as a treatment for difficulty in breathing, they mentioned other placental practices, which were not included in the analysis because these were not among the most frequently-mentioned care practices across sites. Although beyond the scope of this study, future research, such as in-depth qualitative analysis of responses from each individual site, would be appropriate to address these limitations. Likewise, although a single codebook and coding strategy was applied consistently across sites, differences in interviewers, interview techniques, and local terminologies may explain some of the heterogeneity of findings across sites. Despite this, the consistency in findings across sites is encouraging and provides some evidence that recognition of intrapartum-related complications may be easier than other health complications of the newborn, such as infection. However, recognition of the problem is the first step, and appropriate and timely care-seeking is essential, especially for intrapartum-related complications where death can occur minutes after birth if not appropriately managed.

### Conclusions

Intrapartum-related complications account for 23% of all deaths of newborns in Bangladesh (5). This study outlines some of the perceived signs, causes, and treatments for a baby with difficulty in breathing in three sites in Bangladesh. Although there was agreement among respondents in all sites, there were some variations as well. Formative research is essential prior to initiating health programmes to understand the context and develop appropriate health messages and materials. Results from this qualitative study informed the subsequent phase of the abovementioned larger study, which focused on the recognition and response to prolonged labour and breathing difficulties in newborns. In the larger study, we conducted structured interviews using the terms and concepts elicited from the freelisting data described in this article. This allowed for a more detailed analysis of participants’ similarities and differences in their understanding of breathing difficulties in newborns, based on care-giving roles. In another aspect of this project, integrated interviews on illness were conducted with women and family and/or birth attendants present during labour to explore delays in recognition of and care-seeking response to prolonged labour and intrapartum-related complications. Analyses of these data indicated that delays in recognition of and response to prolonged labour resulted from confusion over the onset of labour, power dynamics underlying care provided during labour, preferences for home-births, and practices of ‘waiting for delivery’ (21). Taken together with findings from the current analysis, these findings suggest the need for multi-faceted, integrated approaches to addressing delays in the recognition of and response to intrapartum complications in home-birth settings in Bangladesh. Thus, in the final phase of the project, we integrated findings from the study into the existing birth preparedness/complication readiness programmes across all three sites. We believe others may use the approach described in this study to develop or refine behaviour change interventions to improve recognition of and response to difficulty in breathing at birth in an effort to reduce neonatal mortality in Bangladesh and South Asia.

## ACKNOWLEDGEMENTS

This research was funded by a grant from the Emory Global Health Institute. We would like to thank Daniel Hruschka, Khaleda Jasmin, Stacy Saha, Afroza Khanom Roza, Swateeprova Rahman, Tammanna Gazi, and Shahinoor Akter for their contributions to this research.
